# Mr. Grose, on Hæmorrhœa Petechialis

**Published:** 1801-09

**Authors:** J. H. Grose

**Affiliations:** Winslow


### *To the Editors of the Medical and Phyſical Journal.*

GENTLEMEN,

FROM the readineſs with which every hint at improvement is received into your valuable Journal, I am induced to ſend you the following remarks on an important diſorder, at preſent but imperfectly known, not with a view to promulgate any new diſcovery, but to ſolicit the aſſiſtance and information of thoſe whoſe obſervations have been more extenſive. The hiſtory of the following diſorder, although not wholly novel, has but ſeldom engaged the attention of practitioners; and but a ſmall number of thoſe, who have attentively watched its riſe and influence, have found it deviate from its accuſtomed fatality. According to the variety of ſymptoms that have been diſcovered, it has obtained various denominations, as Hæmorrhœa Petechialis, Petechiæ ſine Febre, and Petechianoſis; but as no accurate pathology has yet been developed, I am inclined to think the petechiæ ſine febre an appropriate appellation.

Authors have not unfrequently deſcribed it as merely ſymptomatic, and in general ariſing from hæmorrhage. Dr. Cullen ſays, "*The petechia has been, by all our noſologiſts, enumerated amongſt the exanthemata; but as, according to the opinion of moſt phyſicians, it is very juſtly held to be always a ſymptomatic affection only, I cannot give it a place.*" As its appearance has, of late years, been more frequent than uſual, and its moſt probable origin traced with ſome degree of accuracy, it has been found chiefly to reſult from univerſal debility, and a general tendency to putrefaction; and that hæmorrhage, ſo far from being a regular diagnoſis of the diſeaſe, has only ſupervened as the petechiæ have accumulated.

The

The firſt inſtance that came within my obſervation was a child four years of age, who had laboured under the complaint about a fortnight. At this time the petechiæ were pretty numerous; the child was exceſſively languid; appetite impaired; the eyes ſunk in the head, and ſome ſmall degree of pyrexia. I had previouſly been informed, by a medical friend in this neighbourhood, that the *coccinella ſeptem punctata*, if adminiſtered in ſcruple doſes, had a ſalutary influence in this complaint; and being well convinced, that unleſs ſome ſpeedy relief could be obtained, the child would inevitably die, I ventured to preſcribe a mixture, with two drachms of cochineal, two drachms of aromatic confection, filled up with the decoct. cort. This was taken in the doſe of two table ſpoons full, three times a day, and regularly perſiſted in during the ſpace of a fortnight, at the expiration of which time, having taken ſix drachms of cochineal, I had the pleaſure to find my little patient reſtored to perfect convaleſcence. During the progreſs of the diſorder, there was little or no tendency to hæmorrhage, and the pulſe between 80 and 95; the legs were frequently ſwelled at night.

One circumſtance, perhaps, it will be neceſſary to mention, the child was very much troubled with ſcorbutic eruptions on the face, hands, and arms, for a conſiderable time previous to the appearance of petechiæ. A generous diet was inſiſted on, and the child partook of animal food and red wine.

The next caſe that occurred was the daughter of Mr. J——, aged five, of a very delicate habit; this little girl had been poorly for a conſiderable time before the petechiæ appeared; the appetite was exceedingly ſmall, and the whole body much waſted: The tongue aſſumed a very dark appearance, and, in fact, there was every reaſon to believe putrefaction would ſhortly take place. Willing to try if the ſole uſe of the coccinella (conſidered as a medicine) would have any ſalutary effect, I preſcribed for the child, powders, containing a ſcruple in each, and directed one to be taken three or four times a day, in a glaſs of port wine. They were regularly continued, and in the ſpace of ten or a dozen days the petechiæ began to diſappear, and her appetite improved conſiderably. In this inſtance there was no tendency to hæmorrhage, nor any diſtreſſing ſymptom but what evidently aroſe from accumulated excitability: the pulſe varied often, and fluſhing heats alternately ſuffuſed the face; but in proportion as the vital powers increaſed in energy, by the cautious exhibition of ſtimuli, the fluſhing heats were removed, the appetite increaſed, and the petechiæ gradually diſappeared; from which we may reaſonably infer, that the complaint, in this inſtance at leaſt, owed its exiſtence to defective excitement.

The

The third and laſt caſe was Mrs. G——; ætat. forty; pale complexion; ſubject to pulmonic complaints, and perpetual dyſpnœa; the legs had been œdematous for ſome time before the petechiæ appeared. She had accuſtomed herſelf to low living, drinking principally water, and eating ſparingly of animal food; pulſe ſcarcely perceptible, and ſhe was peculiarly ſubject to ſyncope. The petechiæ were not numerous, and there appeared ſome large diſcolourations of the ſkin, more reſembling vibices than petechiæ. She was not confined to her room, and occaſionally viſited her friends, but complained of much pain after exerciſe; the petechiæ were of a very bright ſhining purple.

It will be needleſs to particularize every trivial incident. She perſevered in the uſe of coccinella and wine; and the vibices firſt diſappeared, after them the petechiæ. It is now ſome months ſince they left her; and, excepting a few pulmonic ſymptoms, totally abſtracted from the diſeaſe in queſtion, ſhe is tolerably well. How far the influence of coccinella extends in the cure of theſe eruptions, or whether the recovery is to be attributed to the uſe of the wine, I cannot undertake to determine. Of their ſtimulative effects, I cannot ſay I obſerved any demonſtration; probably they may poſſeſs ſome antiputreſcent influence, or a peculiar ſpecific property, that is eſſentially beneficial in the diſorder above mentioned. I have peruſed many narratives, and have generally found their appearance deemed indicative of diſſolution, notwithſtanding the liberal uſe of antiſeptics, fruit, wine, &c. By ſome they are ſuppoſed to ariſe from ſcurvy; probably this may, in part, accelerate their appearance, and if ſo, mineral alteratives, or medicines that determine to the ſkin, may have their ſhare of efficacy. In general, however, the conſtitution will be found ſo much impaired, as to admit of nothing but the tonic plan. It is to be wiſhed, that ſome practitioner, whoſe enquiries have been particularly directed to this dangerous malady, may develope his reſearches, and throw additional light on a ſubject, at preſent but imperfectly underſtood.

I am, &c.

