# Errata

**Published:** 1801-10

**Authors:** 


					p. 234, J. 7, dele iepteni punctata.

				

